# Preparation of Ho^3+^/Tm^3+^ Co-doped Lanthanum Tungsten Germanium Tellurite Glass Fiber and Its Laser Performance for 2.0 μm

**DOI:** 10.1038/srep44747

**Published:** 2017-03-17

**Authors:** Dechun Zhou, Xuemei Bai, Hang Zhou

**Affiliations:** 1 School of Materials Science and Engineering, Changchun University of Science and Technology, Changchun, 130022, China; 2 School of Electronic Information Engineering, Changchun University of Science and Technology, Changchun, 130022, China; 3 School of Electric Power, South China University of Technology, Guangzhou, 510640, China

## Abstract

Ho^3+^/Tm^3+^ co-doped 50TeO_2_-25GeO_2_-3WO_3_-5La_2_O_3_-3Nb_2_O_5_-5Li_2_O-9BaF_2_ glass fiber is prepared with the rod-tube drawing method of 15 μm core diameter and 125 μm inner cladding diameter applied in the 2.0 μm-infrared laser. The 2.0 μm luminescence properties of the core glass are researched and the fluorescence intensity variation for different Tm^3+^ doping concentration is systematically analyzed. The results show that the 2.0 μm luminescence of Ho^3+^ is greatly influenced by the doping concentration ratio of Ho^3+^ to Tm^3+^ and that the maximum fluorescence intensity of the core glass can be obtained and its emission cross section can reach 0.933 × 10^−21^ cm^2^ when the sensitized proportion of holmium to thulium is 0.3 to 0.7 (mol%). Simultaneously, the maximum phonon energy of the core glass sample is 753 cm^−1^, which is significantly lower than that of silicate, gallate and germanate glass and the smaller matrix phonon energy can be conductive to the increase 2.0 μm-band emission intensity. The continuous laser with the maximum laser output power of 0.993 W and 2051 nm -wavelength of 31.9%-slope efficiency is output within the 0.5 m glass fiber and the experiment adopts 1560 nm erbium-doped fiber laser(EDFL) as the pump source and the self-built all-fiber laser. Therefore, the glass fiber has excellent laser characteristics and it is suitable for the 2.0 μm-band laser.

Compared with conventional solid and gas lasers, fiber lasers have many advantages, such as good beam quality, high efficiency, good cooling effect, simple structure and easy operation, and it has become the research focus in the field of laser in recent years^1^,^2^,^3^. Especially, mid-infrared fiber lasers within 2.0 μm-band have broad application prospects and important applications in the long-range laser communications, laser-guided, optoelectronics confrontation, remote sensing, laser surgery of a new generation and other medical and military fields because its laser wavelength is placed in the atmosphere transmission window and it is harmless to the human eyes^4^,^5^,^6^,^7^. Currently, Tm^3+^ and Ho^3+^ are the main active ions to produce 2.0 μm band laser and the radiative transitions of Tm^3+^ ion from energy level ^3^F_4_ to ^3^H_6_ and Ho^3+^ ion from ^5^I_7_ to ^5^I_8_ are one of the effective ways to achieve mid-infrared emission of 2.0 μm-wavelength^8^,^9^.

Typically, the tuning range is from 1.87 μm to 2.16 μm for the solid laser with the single Tm^3+^-doped as the luminescent center, and the fluorescence lifetime of the Tm^3+^ ion is very long, which is conducive to high energy Q-switched laser output. However, higher pumping energy is required to overcome the high threshold power due to the small stimulated emission cross section and quasi-three-level structure for Tm^3+^ at room temperature, which easily leads to excited state absorption of Tm^3+^ and causes inversion consumption^10^,^11^. Compared with Tm^3+^ ions, the stimulated emission cross section of Ho^3+^ is approximately five times as that of Tm^3+^ and the fluorescence lifetime is up to 8ms, which facilitates storage. However, in the single Ho^3+^-doped glass, laser generation efficiency is relatively low because of the non-radiative transitions dominance and Ho^3+^ ions does not correspond to absorption energy levels of 808 nm and 980 nm bands, which lacks of effective pumping source, thereby the single Ho^3+^-doped fiber laser applicability is reduced^12^,^13^. In order to better improve the Ho^3+^ laser performance of 2.0 μm, the sensitized technology is employed usually to improve the luminous efficiency, therefore, researchers began to work on a multi-ions co-doped fiber laser.

The reports of the Tm^3+^-doped, Ho^3+^-doped and Ho^3+^/Tm^3+^ co-doped 2.0 μm luminescent materials focus on the quartz glass, silicate glass, fluoride glass, sulfide glass, tellurite glass and germanate glass^14^,^15^,^16^,^17^,^18^. In 1994, Ghisler *et al*. Of Bern University in Switzerland implemented the laser output of 2.04 μm with 809 nm AlGaAs laser diode pumping Ho^3+^/Tm^3+^ co-doped silica fiber firstly, however, the laser output power was only 5.8 mW. Until 2008, Jackson *et al*. of University of Manchester adopted a 793 nm semiconductor laser to pump Ho^3+^/Tm^3+^ co-doped double-clad silica fibers and obtained a 83-W, 2.015-μm laser output with a slope efficiency of 42%. This is the current highest record for Ho^3+^/Tm^3+^ co-doped laser. In 2009, PFMoulton *et al*. reported a 885 W multimode laser output with a center wavelength of 2.04 μm and a slope efficiency of 49.2%, using a 790 nm diode-pumped double-clad Tm^3+^-doped quartz fiber, which is the current maximum laser output power for the 2.0 μm band Tm^3+^ -doped quartz fiber. Although the output power of 885 W for the 2.0 μm band laser has been realized in Tm^3+^ -doped quartz glass, the quartz glass substrate has the characteristics of high phonon energy, the non-radiative transition energy loss due to multi-phonon relaxation at lower energy level occupies a dominant position, leading to the decrease of radiation quantum efficiency and fluorescence quenching effect, which is disadvantageous to the miniaturization and high gain of fiber lasers and this limits the further improvement of the performance of the 2.0 μm band quartz fiber lasers^19^,^20^. Although fluoride and sulfide substrate glass have lower phonon energy, they have some defects, such as poor chemical stability and mechanical properties, difficult preparation, splice difficulties with standard quartz fiber and low laser damage threshold^21^,^22^. The tellurite glass and the germanate glass have a greater solubility for the rare earth ions, the doping concentration of rare earth ions is high with high refractive index, good chemical stability and thermal stability, which is suitable for drawing the fiber. Instantaneously, its relatively low phonon energy can effectively inhibit the non-radiation transitions of rare earth ions to improve the luminous efficiency for the Ho^3+^/Tm^3+^ co-doped 2.0 μm band^23^,^24^,^25^,^26^. Therefore, the tellurite glass and the germanate glass are ideal gain matrix materials of fiber laser.

In recent years, there are some research reports of 2.0 μm-band spectroscopic properties for the Ho^3+^/Tm^3+^ co-doped germanate glass or tellurite glass, but there are few reports for that of Ho^3+^/Tm^3+^ co-doped lanthanum tungsten germanium tellurite glass fiber. Therefore, the Ho^3+^/Tm^3+^ ions co-doped lanthanum tungsten germanium tellurite glass fiber is prepared with the rod-tube drawing method, based on the Ho^3+^/Tm^3+^ ions co-doped lanthanum tungsten germanium tellurite glass formulations of 50TeO_2_-25GeO_2_-3WO_3_-5La_2_O_3_-3Nb_2_O_5_-5Li_2_O-9BaF_2_, the spectrum properties of prepared fiber is researched, the influences of 2.0 μm luminescence intensity for the different doping concentrations are discussed and the 2051 nm mid-infrared laser output is implemented in the Ho^3+^/Tm^3+^ ions co-doped lanthanum tungsten germanium tellurite glass fiber with self-designed all-fiber laser.

## Experiments

### Glass melting and fiber preparation

A group of lanthanum tungsten germanium tellurite glasses with good physical and chemical properties are selected as the matrix materials for the fiber core and cladding based on the extensive literature^27^,^28^,^29^,^30^,^31^ and experimental work. The formulations for the core and cladding glass are 50TeO_2_-25GeO_2_-3WO_3_-5La_2_O_3_-3Nb_2_O_5_-5Li_2_O-9BaF_2_-xTm^3+^-0.3Ho^3+^ and 39TeO_2_-36GeO_2_- 3WO_3_-5La_2_O_3_-3Nb_2_O_5_-5Li_2_O-9BaF_2_ respectively. The doping concentration x (mol%) of the Tm^3+^ in the core glass material are 0.3, 0.5, 0.7 and 1.0 and the obtained core glass samples are numbered by C_1_, C_2_, C_3_ and C_4_, while the cladding glass sample is numbered by C_5_. All preparation materials of the sample glasses are weighed precisely as the formulations and mixed, which are of analytical reagent grade, stirred evenly and placed in a platinum crucible, then melted for an hour at about 1300 °C in an electric furnace with silicon molybdenum rods heating, after that the clarified molten glass is cast in a preheated steel mold for forming, and then quickly shift to the muffle furnace to anneal precisely, keep the temperature close to the glass transition temperature of T_g_ for 3 hours and then the temperature is down to the room temperature at the speed of 10 °C/h. The quenched glasses are cut, ground and polished and the samples are made as a glass block with both sides polished of 10 mm × 10 mm × 2 mm, a core rod and a cladding tube. The glass block is used for the spectra, while the core rod and cladding tube are assembled as the rod and tube composing, drawing with the rod and tube combination methods.

The drawing technique process with the rod-tube assembly is as follows. Firstly, the core glass rod is put into the cladding glass tube and pulled to a preform with 5 mm-diameter with the optical fiber drawing machine. The preform is placed again in the cladding glass tube for drawing. Eventually, it formed a core diameter of 15 μm, cladding diameter of 125 μm of Ho^3+^/Tm^3+^ ions co-doped lanthanum tungsten germanium tellurite glass fiber is prepared with the twice drawing method. Nitrogen, helium and argon simultaneously protected in the fiber drawing process.

### Performance Testing

The reflection index of the glass bulk sample is measured by the prism coupler. Characteristic temperature is measured with the differential scanning calorimetry (DSC) method and the measurement instrument employs the TAS-100-type thermal analyzer of the Japanese Rigaku Corporation. Absorption spectrum is measured by the Lambda-950-type spectrophotometer with measurement range of 400 nm–2200 nm. Raman spectra measurement employs the inVia Raman microscope of the British Renishaw Company, whose measurement range is from 100 cm^−1^ to 2000 cm^−1^ and the excitation wavelength is 532 nm. Fluorescence spectroscopy measurement adopts the Triax320-type fluorescence spectrometer of French J-Y Company, semiconductor laser pump source of 808 nm is pumped with 2 W-power when measured and the transmitted signal is amplified by the detector, monochromator and lock-in amplifier and the fluorescence decay curve is recorded. The laser output spectrum of the experimental drawn Ho^3+^/Tm^3+^ co-doped lanthanum tungsten germanium tellurite glass fiber is measured by the self-designed all-fiber laser, the 1560 nm erbium-doped fiber laser (EDFL) is selected as the pump source with pump power of 3 W when measured and the output laser after collimation is input to the spectrum analyzer through the attenuator to record the laser spectroscopy. All of the above measurements are carried out at the room temperature if no special instructions.

## Results and Discussions

### Matching and differential thermal analysis of material properties

Fiber core glass and cladding glass refractive index matching is a problem to be considered firstly for the fiber design and the refractive index of the core glass is generally higher than that of the cladding glass^32^. In this study, the reflective index of the Ho^3+^/Tm^3+^ co-doped lanthanum tungsten germanium tellurite core glass and cladding glass samples are given in Table 1. From Table 1, the refractive index of glass samples is increased gradually with the increasing of rare earth ions’ concentration and decreased with the increasing of the GeO_2_, wherein the refractive index difference between the core and the cladding glass is in the range of 0.0268–0.0298, thus the light wave-guide transmission conditions are satisfied in the optical fiber and the optical fiber with the theoretical numerical aperture of 0.3293 or more can be matched.

The differential thermal of the glass samples is measured to study their thermal stability, the temperature range is from 200 °C to 1000 °C. Thermodynamic stability of the glass is commonly measured with the difference between the T_x_ and Tg, that is ΔT = T_x_ − T_g_, where T_g_ is the transition temperature of glass and T_x_ is the crystallization initiation temperature. Greater value of ΔT means better thermal stability of the glass and the specific data are listed in Table 1. It can be seen from Table 1 that ΔT of all the glass samples are greater than 150 °C, which means that the lanthanum tungsten germanium tellurite glass has good thermal stability and the difference of the transition temperatures between the core and cladding glass does not exceed 35 °C to meet the drawing temperature unanimous requests of the lanthanum tungsten germanium tellurite glass fiber and be suitable for drawing. Furthermore, it can also be seen from Table 1 that ΔT increases from 162 °C to 177 °C when GeO_2_ content is increased from 25% to 36%. Experiments show that the increase of the GeO_2_ content in the glass matrix helps improve the thermal stability of lanthanum tungsten germanium tellurite glass and expands the drawing scope of the lanthanum tungsten germanium tellurite glass fiber.

In order to prevent the optical fiber burst due to too much thermal stress and the fiber cladding partial loss because of the weak bond of the core and cladding glass interface during the drawing process, the general requirements for thermal expansion coefficient difference between the core and cladding glass is no more than ± 20 × 10^−7^/°C to ensure the mechanical strength and the geometry structure integrity of the optical fiber^33^. As it can be seen from Table 1 of the thermal expansion coefficient of α-data that the maximum difference of the thermal expansion coefficients between the core and cladding glass is 11.7 × 10^−7^/°C, which is in full compliance with the requirements of fiber drawing. The above data analysis shows that the performance matching of the core and cladding glass is good and the experimental designed core and cladding matrix glass is very suitable for the preparation of glass fiber.

### Analysis of absorption spectrum

**Figure 1:**
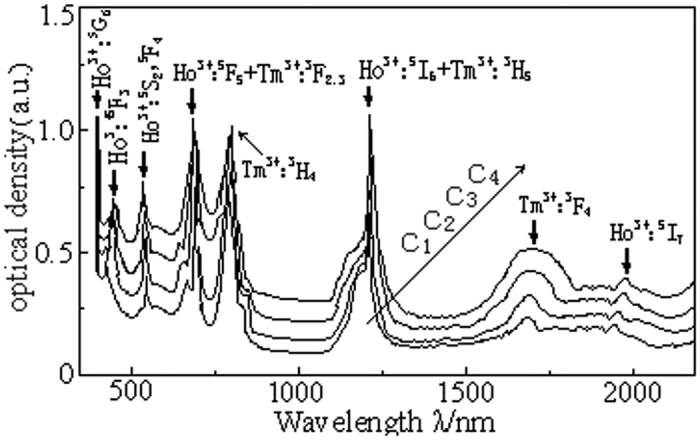
The absorption spectra of the Ho^3+^/Tm^3+^ co-doped lanthanum tungsten germanium tellurite glass.

Figure 1 shows the absorption spectrum in the range of 400 nm–2200 nm of the Ho^3+^/Tm^3+^ co-doped lanthanum tungsten germanium tellurite core glass sample and the corresponding excited state energy level has been marked in the figure. It can be seen from Fig. 1 that there are six main absorption bands of the Ho^3+^ ion in the glass sample and the wavelengths are located at the peak of 1953 nm, 1178 nm, 646 nm, 539 nm, 460 nm and 420 nm, which correspond to the absorption transition of Ho^3+^ ion from the ground level of ^5^I_8_ to the excited state levels of ^5^I_7_, ^5^I_6_, ^5^F_5_, (^5^S_2_, ^5^F_4_), ^5^F_3_ and ^5^G_6_, while the energy levels absorption of the other excited states have been overshadowed by the matrix absorption. There are four important absorption bands of the Tm^3+^ ion in the glass sample, the wavelengths are located at the 1680 nm, 1210 nm, 793 nm ad 687 nm, which correspond to the absorption transition of Tm^3+^ from the ground level of ^3^H_6_ to the excited state levels of ^3^F_4_, ^3^H_5_, ^3^H_4_ and ^3^F_2.3_, while the energy levels absorption of the other excited states have been overshadowed by the matrix absorption. The energy level of Tm^3+^:^3^F_4_ which transmits energy has a very obvious, alone absorption peak, which lays a good foundation for the energy level of Ho^3+^:^5^I_7_ which receives energy to produce 2.0 μm-laser. In addition, since there also is an alone obvious absorption peak in the vicinity of 793 nm-wavelength for the Tm^3+^, the drawn fiber glass sample can choose a lower-cost 808 nm- wavelength laser pump source of LD for the effective pumping.

It can also be observed from Fig. 1 that the intensity of the absorption peaks gradually increase and the absorption peaks gradually strengthen of Ho^3+^ as Tm^3+^ doping concentration increases. The absorption peak is strongest when Tm^3+^ ion concentration is 1.0 mol%. The intensity of the absorption is maximum when the Ho^3+^/Tm^3+^ concentration ratio (mol%) is 0.3 to 1.0, while the each absorption peak shape and intensity of Ho^3+^ have no significant changes, which indicates that the different doping ratios for Ho^3+^ to Tm^3+^ has little influences for the 2.0 μm absorption of Ho^3+^ ion. In addition, the spectrum shape and the absorption peak position of the Ho^3+^/Tm^3+^ co-doped lanthanum tungsten germanium tellurite glass are similar to those of the other oxyfluoride and fluorine germanate glass matrix reported in the literature^16^,^17^.

### Fluorescence spectroscopy and Raman spectroscopy

**Figure 2:**
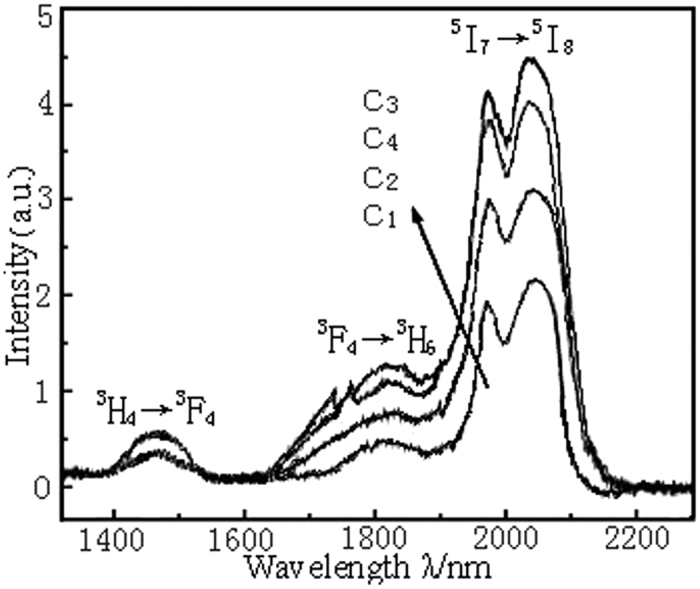
The fluorescence spectra of Ho^3+^/Tm^3+^ co-doped lanthanum tungsten germanium tellurite core glass.

Figure 2 is the fluorescence spectra for the Ho^3+^/Tm^3+^ co-doped lanthanum tungsten germanium tellurite core glass pumped at the 808 nm-wavelength LD. From Fig. 2, there are three fluorescence emission bands of 1.47 μm, 1.80 μm and 2.0 μm in the range of 1300 nm–2200 nm, which correspond to the energy level transitions from ^3^H_4_ to ^3^F_4_, from ^3^F_4_ to ^3^H_6_ of the Tm^3+^ ion and from ^5^I_7_ to ^5^I_8_ of the Ho^3+^ ion. It can be seen clearly from Fig. 2 that when the doping concentration of the Ho^3+^ remains 0.3 mol%, the intensity change of 1.47 μm-fluorescence produced by the ^3^H_4_ to ^3^F_4_ energy level transition of the Tm^3+^ is smaller as the increasing of the Tm^3+^ concentration and the transition peak of the 1.80 μm caused by the ^3^F_4_ to ^3^H_6_ energy level transition of the Tm^3+^ is in a strengthening trend, while the intensity of 2.0 μm-fluorescence generated by ^5^I_7_ to ^5^I_8_ energy level transition of Ho^3+^ increases sharply. Figure 1 of the absorption spectra shows that Ho^3+^ ions has no significant absorption for the 808 nm pumping light, and therefore 2.0 μm luminescence is only from Tm^3+^ energy transfer, which indicates that there exists a strong energy transfer process between Tm^3+^(^3^F_4_) and Ho^3+^(^5^I_7_). The fluorescence intensity reaches maximum in the vicinity of 2.0 μm for the Ho^3+^ of sample C_3_ when the doping concentration of Tm^3+^ ions reaches 0.7 mol%. However, when Tm^3+^-doped concentration reaches 1.0 mol%, the sensitization of Ho^3+^ decreases due to the Tm^3+^ concentration quenching effect, the anti-cross relaxation effect of ions and the reverse energy transfer from Ho^3+^(^5^I_7_) to Tm^3+^(^3^F_4_), thus, the 2.0 μm-fluorescence intensity of C_4_ sample begins to decrease significantly. This shows that there exists an optimal sensitized ratio of 0.3 to 0.7 for the Ho^3+^/Tm^3+^ ions co-doped of lanthanum tungsten germanium tellurite glass matrix and the fluorescence spectra intensity decreases significantly when the sensitized ratio exceeds it.

**Figure 3:**
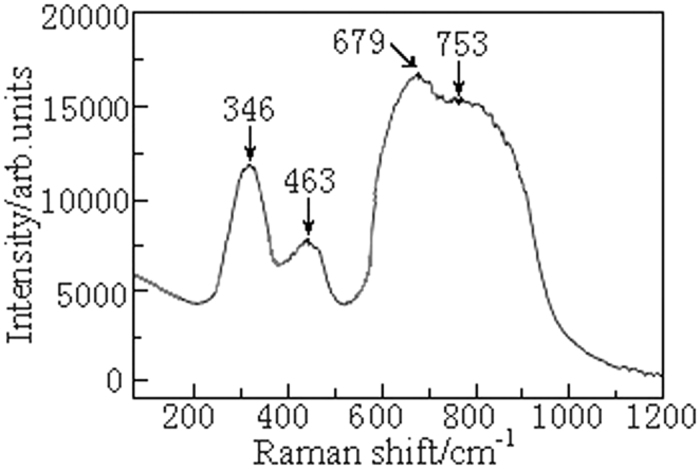
The Raman spectroscopy of the Ho^3+^/Tm^3+^ co-doped lanthanum tungsten germanium tellurite glass.

Figure 3 shows the Raman spectra of glass samples C_3_ and it can be seen from the figure that there are four Raman peaks located at the positions of 346 cm^−1^, 463 cm^−1^, 679 cm^−1^, and 753 cm^−1^. Raman characteristic peak of 346 cm^−1^ corresponds to W-O-W bending vibration in the octahedron of [WO_6_], Raman characteristic peak of 463 cm^−1^ corresponds to the symmetric bending vibration of Te-O-Te bond and stretching vibration of O-Ge-O in [GeO_4_], Raman characteristic peak of 679 cm^−1^ belongs to the biconical stretching vibration in [TeO_4_], and Raman characteristic peak of 753 cm^−1^ corresponding to the maximum phonon energy, belongs to the stretching vibration of O-Ge-O bond in [GeO_6_] and the tripartite cone stretching vibration in [TeO_3_] or [TeO_3+1_]. Therefore, the maximum phonon energy of the glass sample C_3_ is 753 cm^−1^ and it is significantly lower than that of the silicate glass, gallate glass and germanate glass^34^,^35^,^36^. As for the Ho^3+^ and Tm^3+^ co-doped 2.0 μm-emitting, such low phonon energy can reduce the non-radiative transition probability of multi-phonon, which helps to increase the 2.0 μm-band emission intensity.

### Calculation and comparative analysis for the spectral parameters

Judd-Ofelt theory is usually employed to calculate the spectral strength parameters (Ω_2_, Ω_4_, Ω_6_) of the rare earth ions in different glass matrix to analyze the ordering of the glass structure, symmetry of rare earth ions ligand field and so on. It is generally believed that the greater Ω_2_ means the lower symmetry and stronger covalence of the material rare earth ions ligand field, the greater Ω6 indicates the weaker covalence of the glass rare earth ions and anions, while the ratio of Ω_4_ to Ω_6_ determines the spectral quality of the matrix glass^22^,^37^.

According to the absorption spectroscopy obtained by experiments, the J-O theory is employed to calculate the line parameters of Ho^3+^ ions in the sample C_3_ and they are compared with the spectral parameters of different glass matrix with the values listed in Table 2. Table 2 shows that the value of Ω_2_ of Ho^3+^ in the lanthanum tungsten germanium tellurite glass is 6.13 × 10^−20^ cm^2^, much larger than that of the germanate, tellurite and silicate glass, which indicates that the covalence of the glass is relatively stronger, the symmetry of the glass rare earth ions and ligand field is lower to be excited easily. While, the value of Ω_6_ of Ho^3+^ in the lanthanum tungsten germanium tellurite glass is 1.39 × 10^−20^ cm^2^, greater than that of silicate, gallate and germanate, but lower than that of tellurite and fluorophosphate glass, which indicates that non-bridging oxygen ions of the lanthanum tungsten germanium tellurite glass is less than that of the tellurite glass and fluorophosphate glass, therefore, the stability of the system is preferably better than that of the tellurite glass and fluorophosphate glass. The spontaneous emission transition probability from ^5^I_7_ energy level to ^5^I_8_ energy level for the Ho^3+^ in the glass sample C_3_ of Ar is calculated as 259.13 s^−1^ with the calculated spectral intensity parameter values, which is close to the parameter data reported in the literature^22^ and has higher spontaneous radiative transition probability compared with the other fluorophosphate, gallate, germanate and silicates listed in Table 2, meaning that the Ho^3+^/Tm^3+^ co-doped lanthanum tungsten germanium tellurite glass can produce stronger 2.0 μm-fluorescence emission.

**Figure 4:**
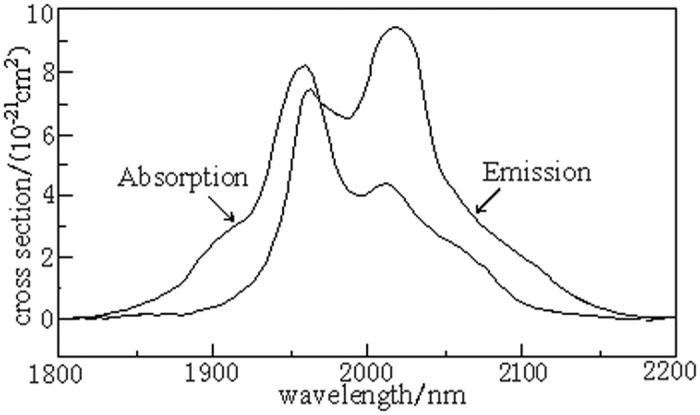
2.0 μm-absorption and emission cross section for Ho^3+^ of lanthanum tungsten germanium tellurite glass.

The doped rare earth ions absorption cross-section of 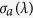 and the stimulated emission cross section of 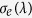 of the Lanthanum tungsten germanium tellurite glass can be calculated from the absorption spectra measured in the Fig. 1 and the Lambert-Beer law and McCumber theory.


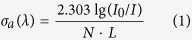



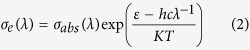


where, *I*_0_ is the incident light intensity, *I* is the transmitted light intensity, *N* is the number of the doped rare earth ions per unit volume, *L* is the thickness of the sample (which is 2 mm), *ε* is the free energy of the rare earth ions transition from the ground state to the excited state, λ is the emission light wavelength, k is the Boltzmann constant, h is Planck’s constant, c is the light speed and T is the sample temperature. Figure 4 shows the absorption and emission cross section of Ho^3+^ near 2.0 μm. Figure 4 shows that the maximum absorption cross section of the Ho^3+^ ions in the C_3_ glass sample is located at the 1953 nm-wavelength of 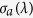 = 8.109 × 10^−21^ cm^2^ and the maximum emission cross section is located at 2051 nm-wavelength of 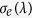 = 0.933 × 10^−21^ cm^2^. It can be seen from Fig. 4 and Table 2 that C_3_ glass sample have the largest absorption cross section and the largest emission cross section, which are bigger than those of tellurite glass and are nearly double of the other fluorophosphate, gallate and germanate glasses. This is determined by the higher refractive index of the lanthanum tungsten germanium tellurite glass because the glass substrate with a larger refractive index can produce higher spontaneous emission transition probabilities and larger emission cross sections. Therefore, C_3_ glass sample is expected to become an important matrix material of 2.0 μm fiber laser.

### Testing and analysis of the laser performances

The laser output characteristics of the Ho^3+^/Tm^3+^ co-doped lanthanum tungsten germanium tellurite glass fiber is tested and analyzed using self-built all-fiber laser and the pumping source selects erbium-doped fiber lasers with the output wavelength of 1560 nm. In this study, a multimode fiber grating with 90%-reflectance is employed as a pre-mirror and a single-mode fiber grating with 10%-reflectance is employed as a post-mirror, thereby, a laser resonator is constituted. The gain fiber of the seed source is the Ho^3+^/Tm^3+^ co-doped lanthanum tungsten germanium tellurite glass fiber of C_3_ drawn in the experiment. The fiber grating is written directly with a phase mask method at a length of 0.5 m of Ho^3+^/Tm^3+^ co-doped lanthanum tungsten germanium tellurite glass fiber^39^. Since the diameter of the Ho^3+^/Tm^3+^ co-doped lanthanum tungsten germanium tellurite glass fiber is different from the diameter of erbium-doped fiber laser pigtail, a tapered fiber is used in the experiment in order to ensure high efficient welding couple, and the fusion technology is employed to achieve the low-loss connections between the optical fibers and the high efficiency pump laser power delivery, thus ensuring high pump light coupling efficiency.

**Figure 5:**
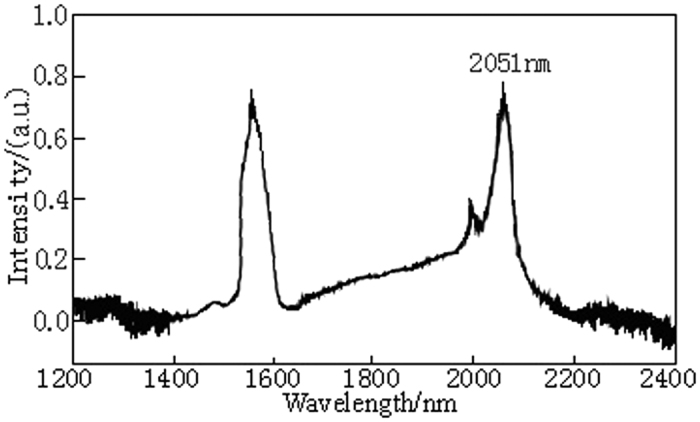
The output laser spectrum of the Ho^3+^/Tm^3+^ co-doped lanthanum tungsten germanium tellurite glass fiber laser.

**Figure 6:**
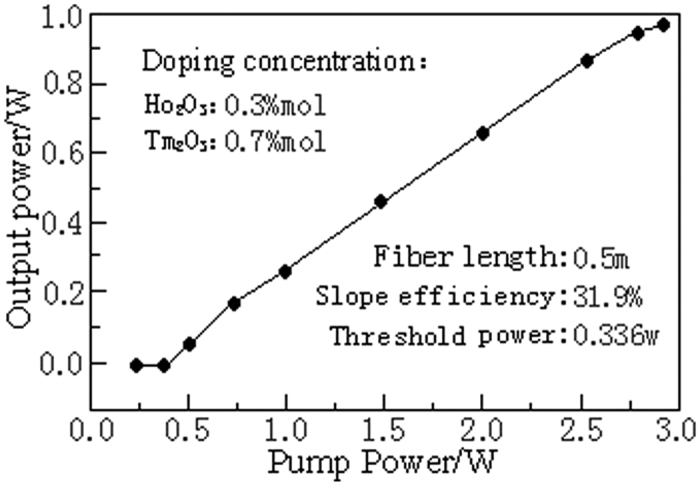
The output power curve of the Ho^3+^/Tm^3+^ co-doped lanthanum tungsten germanium tellurite glass fiber laser.

The EDFL pumping power is 3 W and the output laser of the Ho^3+^/Tm^3+^ co-doped lanthanum tungsten germanium tellurite glass fiber laser is collimated, input to the spectrum analyzer by the attenuator and measured by the laser spectroscopy. The output laser spectrum is shown in Fig. 5. As can be seen from Fig. 5 that the Ho^3+^/Tm^3+^ co-doped lanthanum tungsten germanium tellurite glass fiber can produce laser with a wavelength of 2051 nm. The relationship curve between the laser output power and the into-fiber pumping power measured by the optical power meter is shown in Fig. 6. Experimental results show that there is laser output when the input pumping threshold power reaches 0.336 W. When the pumping power is 2.97 W, the maximum output power generated by the laser is 0.993w with the slope efficiency of 31.9%. It can be seen from Fig. 6 that there is a good linear relationship between the laser output power and input pumping power and there is no saturation phenomenon when the maximum output power of the laser reaches 0.993w. The fiber in the article is compared with the Ho^3+^/Tm^3+^ double-doped laser fiber of similar data and attribute and its laser output power and slope efficiency are higher than those of the fiber reported in the literature^40^,^41^,^42^,^43^, showing that the optical fiber with the Ho^3+^/Tm^3+^ co-doped makes full use of the photo-sensitivity of Tm^3+^ and on the other hand the doping concentration of Ho^3+^ ions is reduced, thereby reducing the laser re-absorption. Therefore, high concentration of Tm^3+^ and Ho^3+^ co-doped lanthanum tungsten germanium tellurite glass fiber can achieve higher laser output power and slope efficiency, which has excellent laser characteristics.

## Conclusions

The Ho^3+^/Tm^3+^ co-doped lanthanum tungsten germanium tellurite glass fiber with the excellent thermal stability and optical properties is prepared. Studies have found that 2.0 μm-band fluorescence emission intensity is maximum with its emission cross section of 0.933 × 10^−21^ cm^2^ when the molar concentration ration of Ho^3+^ to Tm^3+^ reaches 0.3:0, 7 in the lanthanum tungsten germanium tellurite core glass with system of 50TeO_2_-25GeO_2_-3WO_3_-5La_2_O_3_-3Nb_2_O_5_-5Li_2_O-9BaF_2_. This is the highest emission cross section of the Ho^3+^/Tm^3+^ ions co-doped germanium tellurite glass in current reports and the emission cross section of the glass is nearly doubled as that of the other fluorophosphate, gallate and germanate glass. In addition, the maximum phonon energy of the lanthanum tungsten germanium tellurite glass samples is 753 cm^−1^, which is significantly lower than that of the silicate, gallate and germanate glasses. As for the 2.0 μm luminous of the Ho^3+^ and Tm^3+^ co-doped glass, the results show that the lower phonon energy can reduce the non-radiative transition probability of multi-phonons, which is conducive to increase 2.0 μm-band emission intensity.

A 1560 nm-pumped 2.0 μm-band Ho^3+^/Tm^3+^ co-doped lanthanum tungsten germanium tellurite glass fiber laser is self-built during laser performance testing. The 2051 nm-laser output is achieved with this laser. Laser testing finds that the laser threshold power is 0.336 W at this length. When the pumping power is 2.97 W, the maximum output power of the laser is 0.993w and the slope efficiency is 31.9%. Comparative analysis of the drawn Ho^3+^/Tm^3+^ ions co-doped lanthanum tungsten germanium tellurite glass fiber with the tellurite and germanate glasses of similar properties find that high concentrations of Tm^3+^ and Ho^3+^ co-doped lanthanum tungsten germanium tellurite glass fiber can get higher laser output power and slope efficiency, which has excellent laser characteristics and is an ideal mid-infrared laser material.

## Additional Information

**How to cite this article**: Zhou, D. *et al*. Preparation of Ho^3+^/ Tm^3+^ Co-doped Lanthanum Tungsten Germanium Tellurite Glass Fiber and Its Laser Performance for 2.0µm. *Sci. Rep.* **7**, 44747; doi: 10.1038/srep44747 (2017).

**Publisher's note:** Springer Nature remains neutral with regard to jurisdictional claims in published maps and institutional affiliations.

## Figures and Tables

**Table 1:** The refractive index and characteristics temperature of the glass samples.

*No. of samples*	*C*_*1*_	*C*_*2*_	*C*_*3*_	*C*_*4*_	*C*_*5*_
*The refractive index*	2.0361	2.0369	2.0383	2.0391	2.0093
T_g_/°C	501	503	506	510	519
T_X_/°C	663	667	669	673	696
T_X_−T_g_/°C	162	164	163	163	177
α × 10^−7^/°C	102.7	106.3	110.9	108.6	99.2

**Table 2:** The comparison of the spectra parameters for Ho^3+^ in different glass matrix.

*sample*	*Ω2/10−20* *cm*^2^	*Ω*_*4*_*/10*^*−20*^ *cm*^2^	*Ω*_*6*_*/10*^*−20*^ *cm*^2^	*λ*_*P*_/*nm*	A_*r*_/*s*^*−1*^	*τ*_*r*_*/ms*	*σ*_*e*_*/10*^*−20*^ *cm*^2^
Silicate^38^	3.60	3.01	0.61	2040	61.65	16.22	0.70
Fluorphosphate^38^	1.92	2.18	1.71	2050	69.21	14.45	0.56
Galliumsalts^38^	4.77	2.18	1.22	2055	69.53	14.38	0.38
Germanate^38^	3.52	2.78	1.24	2035	73.33	13.64	0.51
Tellurate^22^	5.26	2.28	2.18	2027	257.50	3.90	0.915
C_3_(this experiment)	6.13	3.51	2.39	2051	259.13	3.86	0.933
